# Interactions between serum urate-associated genetic variants and sex on gout risk: analysis of the UK Biobank

**DOI:** 10.1186/s13075-018-1787-5

**Published:** 2019-01-09

**Authors:** Ravi K. Narang, Ruth Topless, Murray Cadzow, Greg Gamble, Lisa K. Stamp, Tony R. Merriman, Nicola Dalbeth

**Affiliations:** 1 Department of Medicine, Faculty of Medical and Health Sciences, University of Auckland, 85 Park Road, Grafton, Auckland, 1023 New Zealand; 2 Department of Biochemistry, University of Otago, 710 Cumberland Street, Dunedin, 9012 New Zealand; 3 Department of Medicine, University of Otago, Christchurch, 2 Riccarton Avenue, Christchurch, 8140 New Zealand

**Keywords:** Gout, Genetics, Urate

## Abstract

**Background:**

Sex-specific differences in the effect of genetic variants on serum urate levels have been described. The aim of this study was to systematically examine whether serum urate-associated genetic variants differ in their influence on gout risk in men and women.

**Methods:**

This research was conducted using the UK Biobank Resource. Thirty single nucleotide polymorphisms (SNPs) associated with serum urate were tested for their association with gout in men and women of European ancestry, aged 40–69 years. Gene-sex interactions for gout risk were analysed using an interaction analysis in logistic regression models.

**Results:**

Gout was present in 6768 (4.1%) men and 574 (0.3%) women, with an odds ratio (95% confidence interval) for men 13.42 (12.32–14.62) compared with women. In men, experiment-wide association with gout was observed for 21 of the 30 serum urate-associated SNPs tested, and in women for three of the 30 SNPs. Evidence for gene-sex interaction was observed for *ABCG2* (rs2231142) and *PDZK1* (rs1471633), with the interaction in *ABCG2* driven by an amplified effect in men and in *PDZK1* by an absence of effect in women. Similar findings were observed in a sensitivity analysis which excluded pre-menopausal women. For the other SNPs tested, no significant gene-sex interactions were observed.

**Conclusions:**

In a large population of European ancestry, *ABCG2* and *PDZK1* gene-sex interactions exist for gout risk, with the serum urate-raising alleles exerting a greater influence on gout risk in men than in women. In contrast, other serum urate-associated genetic variants do not demonstrate significant gene-sex interactions for gout risk.

**Electronic supplementary material:**

The online version of this article (10.1186/s13075-018-1787-5) contains supplementary material, which is available to authorized users.

## Background

Sex differences in the epidemiology, clinical characteristics, and risk factors for gout have been reported. Prevalence among gout is higher in men [1], and women with gout are more likely to be older, have co-morbidities, and be on diuretics compared with men with gout [2, 3]. Similar findings have also been noted with respect to serum urate levels, with men having higher levels than women with these differences decreasing with advancing age [4].

Raised serum urate is the major risk factor for gout [5, 6]. The heritability of serum urate is estimated at 40–70% [7–9] and over the past decade genome-wide association studies (GWAS) have identified single nucleotide polymorphisms (SNPs) associated with serum urate and gout [10–16]. Sex-specific analysis of genotypes associated with serum urate and gout have also been examined. The magnitude of effect of the *ABCG2* variant appears to be greater in men than women for both serum urate and gout risk [13, 16]. Conversely, the *SLC2A9* variant has been shown to exert greater influence for serum urate in pre-menopausal women compared with post-menopausal women and with men [17]. It is unclear whether other serum urate-associated genetic variants display sex-specific differences for gout risk.

The aim of this study was to systematically examine whether serum urate-associated genetic variants differ in their influence on gout risk in men and women.

## Methods

This research was conducted using the UK Biobank Resource (approval number 12611). Participants of European ancestry who were aged 40–69 years and with genome-wide genotypes were included in this study. Exclusion criteria included mismatch between self-reported sex and genetic sex, genotyping quality control failure, related individuals, and participants aged 70 years and over. Gout was defined using a validated definition of self-report of gout or urate-lowering therapy use (including allopurinol, febuxostat, and sulphinpyrazone, and participants must not have a hospital diagnosis of leukaemia or lymphoma based on the International Classification of Diseases, Tenth Revision codes C81-C96) [18]. For participants who did not meet the gout definition, further exclusion criteria included prescriptions for corticosteroids, non-steroidal anti-inflammatory drugs or probenecid. Medication use, co-morbidities, alcohol, smoking status, and menopausal status data were collected via self-report.

UK Biobank samples were genotyped using an Axiom array (820,967 markers, Affymetrix, Santa Clara, CA, USA) and imputed to approximately 73.3 million SNPs using SHAPEIT3 and IMPUTE2 with a combined UK10K and 1000 Genomes reference panel. We analysed the 30 SNPs associated with serum urate reported by Kottgen et al. [10] in the large (> 140,000 European participants) Global Urate Genetics Consortium GWAS.

Data were analysed using IBM SPSS Statistics 25 software. Baseline characteristics are summarised using standard descriptive statistics including means, standard deviations (SD), and number and percent, and were tested using unpaired *t* tests or chi-squared tests where appropriate. Logistic regression of the 30 SNPs with gout as the dependent variable in men and women was performed. The primary analysis calculated association with gout based on the proportion of participants with at least one effect allele present. The number of effect alleles was included in the secondary analysis. Gene-sex interactions for gout risk were analysed using logistic regression models that included an SNP by sex interaction term. Women with no effect allele were used as the referent group in the stratified logistic regression analysis. Age, body mass index, renal failure, and diuretic use were included as variables in all models. We also performed a sensitivity analysis which excluded pre-menopausal women. Data are reported at experiment-wide significance (*P* < 0.0017).

## Results

### Clinical features of participants

Data including genome-wide genotypes were available for 359,876 participants. Baseline characteristics are shown in Table 1 with genotype frequencies of the 30 serum urate-associated SNPs shown in Additional file 1 (Table S1). There were 188,221 (53.2%) women, of whom 142,272 (75.6%) were post-menopausal. Overall, there were 7342 (2.0%) participants fulfilling the study criteria for gout. Gout was present in 6768 (4.1%) men and 574 (0.3%) women, with an odds ratio (OR) of 13.42 (95% confidence interval (CI) 12.32–14.62) for men compared with women. Women with gout were older (mean ± SD, 61.8 ± 5.9 years vs 59.8 ± 6.9 years, *P* = 2.90 × 10^−11^), had a higher body mass index (BMI; 32.3 ± 6.6 kg/m^2^ vs 30.6 ± 4.8 kg/m^2^, *P* = 7.13 × 10^−16^), higher diuretic use (35.9% vs 14.8%, *P* = 6.05 × 10^−39^), and a higher prevalence of renal failure (3.1% vs 1.3%, *P* = 5.59 × 10^−4^) compared with men with gout.

**Table 1:** Baseline characteristics of participants according to overall group and sex

	All patients *n* = 359,876		Women *n* = 188,221		Men *n* = 171,655		Men vs women (gout cases) *P*
	Control *n* = 352,534	Gout *n* = 7342	Control *n* = 187,647	Gout *n* = 574	Control *n* = 164,887	Gout *n* = 6768	
Age, years (SD)	56.9 (8.0)	60.0 (6.9)	56.8 (7.9)	61.8 (5.9)	57.0 (8.1)	59.8 (6.9)	2.90 × 10^−11^
BMI, kg/m^2^ (SD)	27.2 (4.6)	30.7 (4.9)	26.8 (5.0)	32.3 (6.6)	27.6 (4.1)	30.6 (4.8)	7.13 × 10^−16^
Smoker, *n* (%)*	35,768 (10.2%)	661 (9.0%)	16,006 (8.6%)	59 (10.3%)	19,762 (12.0%)	602 (8.9%)	0.34
Alcohol frequency, *n* (%)*							
Daily or almost daily	74,318 (21.1%)	2475 (33.7%)	31,696 (16.9%)	90 (15.7%)	42,622 (25.9%)	2385 (35.3%)	8.12 × 10^−102^
One to four times a week	85,226 (24.2%)	2026 (27.6%)	40,499 (21.6%)	86 (15.0%)	44,727 (27.1%)	1940 (28.7%)	
Once or twice a week	93,145 (26.4%)	1668 (22.7%)	49,718 (26.5%)	130 (22.7%)	43,427 (26.4%)	1538 (22.7%)	
Infrequent**	76,172 (21.6%)	838 (11.4%)	50,509 (26.9%)	178 (31.0%)	25,663 (15.5%)	660 (9.8%)	
Never	23,442 (6.7%)	327 (4.5%)	15,108 (8.1%)	89 (15.5%)	8334 (5.1%)	238 (3.5%)	
Diuretic use, *n* (%)*	28,722 (8.1%)	1210 (16.5%)	16,031 (8.5%)	206 (35.9%)	12,691 (7.7%)	1004 (14.8%)	6.05 × 10^−39^
Co-morbidities, *n* (%)*							
Hypercholesterolaemia	42,929 (16.7%)	2056 (28.2%)	18,322 (13.4%)	180 (31.6%)	24,607 (20.5%)	1876 (27.9%)	0.06
Hypertension	89,575 (34.9%)	4161 (57.0%)	42,152 (30.8%)	364 (63.9%)	47,423 (39.5%)	3797 (56.4%)	5.95 × 10^−4^
Peripheral vascular disease	616 (0.2%)	10 (0.1%)	358 (0.3%)	4 (0.7%)	258 (0.2%)	6 (0.1%)	1.47 × 10^−4^
Angina	11,265 (4.4%)	662 (9.1%)	3603 (2.6%)	59 (10.4%)	7662 (6.4%)	603 (9.0%)	0.27
Myocardial infarction	8261 (3.2%)	521 (7.1%)	1604 (1.2%)	25 (4.4%)	6657 (5.5%)	496 (7.4%)	0.01
Heart failure	196 (0.1%)	44 (0.6%)	73 (0.1%)	5 (0.9%)	123 (0.1%)	39 (0.6%)	0.38
Arrhythmia	1952 (0.8%)	71 (1.0%)	950 (0.7%)	3 (0.5%)	1002 (0.8%)	68 (1.0%)	0.26
Stroke	4733 (1.8%)	254 (3.5%)	1895 (1.4%)	31 (5.4%)	2838 (2.4%)	223 (3.3%)	0.01
Transient ischaemic attack	1343 (0.5%)	59 (0.8%)	623 (0.5%)	5 (0.9%)	720 (0.6%)	54 (0.8%)	0.85
Renal failure	443 (0.1%)	108 (1.5%)	222 (0.1%)	18 (3.1%)	221 (0.1%)	90 (1.3%)	5.59 × 10^−4^
Diabetes mellitus	16,108 (6.3%)	1005 (13.8%)	5846 (4.3%)	110 (19.3%)	10,262 (8.5%)	895 (13.3%)	0.01

*BMI* body mass index, *SD* standard deviation

*Smoking status, alcohol frequency, medication use and co-morbidity data collected via self-report

** Infrequent alcohol frequency defined as one to three times a month, or special occasions only

### Association with gout of serum urate-associated SNPs in men and women

**Fig. 1:**
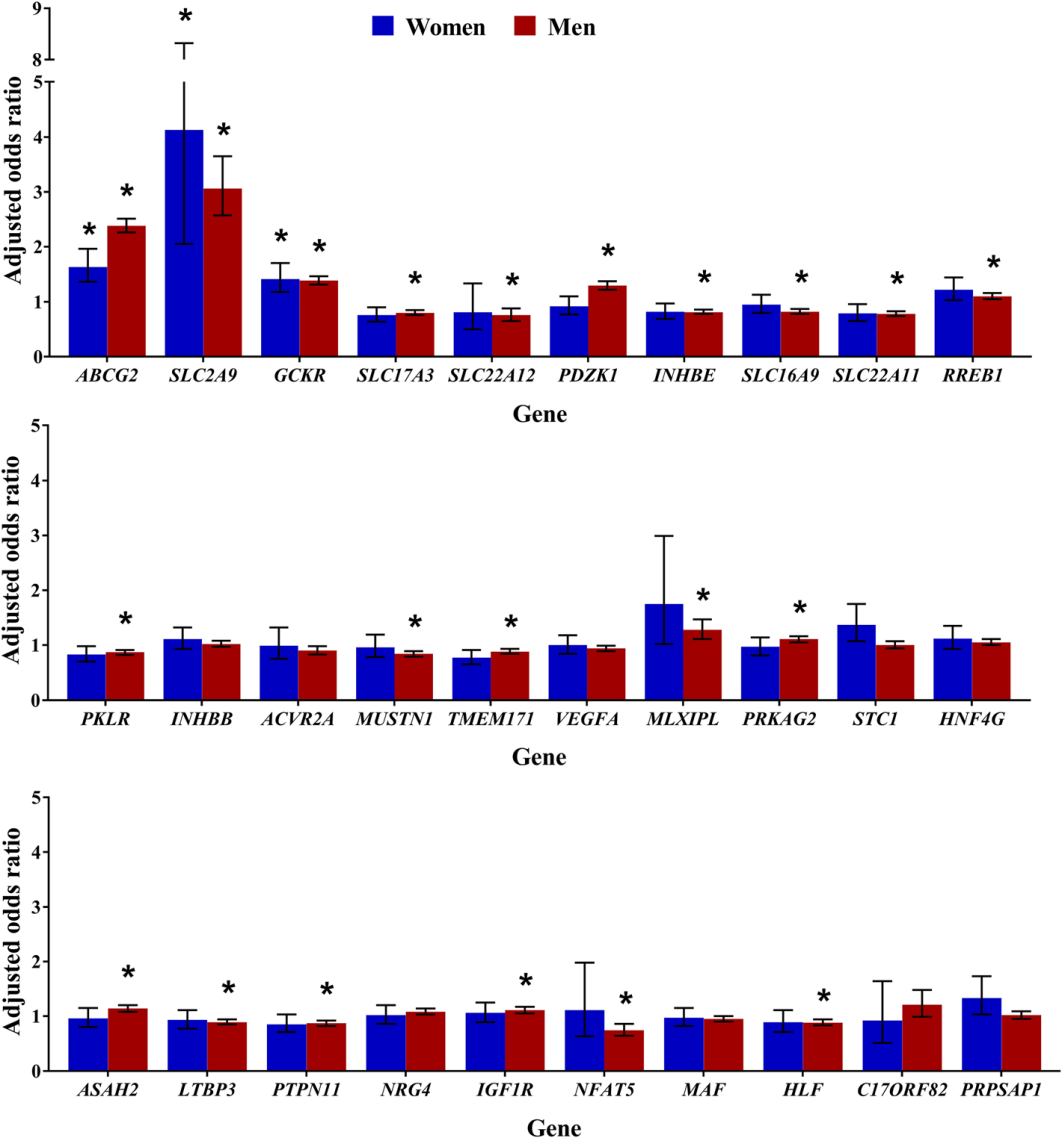
Association analysis of 30 serum-urate associated single-nucleotide polymorphisms for gout. Data are adjusted for age, body mass index, diuretic use, and renal failure. *Experiment-wide significance at *P* < 0.0017

In the entire group, association with gout at experiment-wide significance was observed for 21 of the 30 serum urate-associated SNPs tested (Fig. 1). In men, experiment-wide association was observed for the same 21 SNPs, and in women this association was seen for three of the 30 SNPs: *SLC2A9* (rs12498742), *ABCG2* (rs2231142), and *GCKR* (rs1260326, Fig. 1).

Similar findings were observed when calculating allelic odds ratios based on the number of effect alleles present (Additional file 1: Tables S2 and S3). Association with gout at experiment-wide significance was observed for 22 SNPs in the entire group. In men, experiment-wide association was observed for the same 22 SNPs, and in women this association was seen for four of the 30 SNPs: *SLC2A9* (rs12498742), *ABCG2* (rs2231142), *GCKR* (rs1260326), and *MLXIPL* (rs1178977).

*SLC2A9* (rs12498742) and *ABCG2* (rs2231142) variants exerted the largest effect on gout risk in the group overall (OR for gout 3.07 (95% CI 2.59–3.64), *P* = 8.30 × 10^−39^ and 2.26 (2.15–2.37), *P* = 1.64 × 10^−233^, respectively; Fig. 1). For the *ABCG2* variant a higher risk in men was observed compared with women (OR for gout in men 2.38 (95% CI 2.26–2.51), *P* = 3.72 × 10^−235^, and in women 1.63 (1.36–1.96), *P* = 1.19 × 10^−7^), whilst for the *SLC2A9* variant the risk was not statistically different between sexes as demonstrated by overlapping 95% CIs (OR for gout in men 3.06 (95% CI 2.57–3.65), *P* = 6.71 × 10^−36^, and in women 4.13 (2.05–8.32), *P* = 7.06 × 10^−5^, Fig. 1).

### SNP-sex interaction analysis

**Fig. 2:**
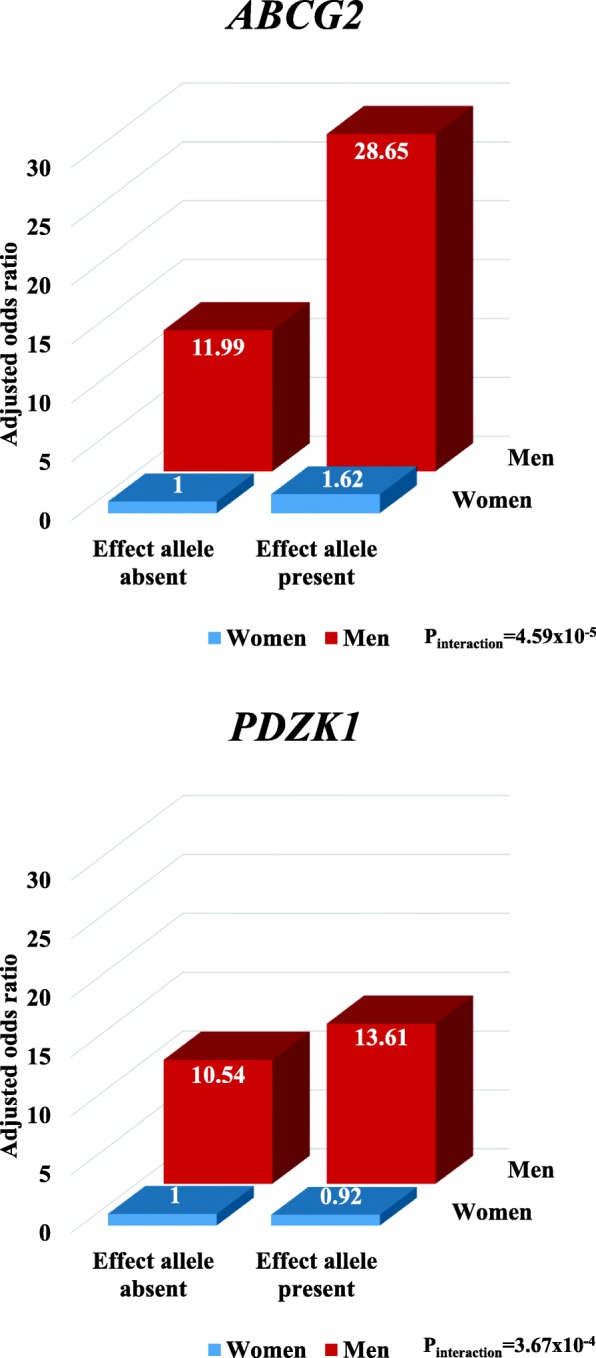
Association and interaction between serum urate-associated genetic variants (for *ABCG2* and *PDZK1*) and sex for gout risk according to effect allele presence. Data are adjusted for age, body mass index, renal failure, and diuretic use. Experiment-wide significance is defined as *P* < 0.0017

Evidence for gene-sex interaction was observed for *ABCG2* (rs2231142) and *PDZK1* (rs1471633), with the interaction at *ABCG2* driven by a larger effect in men, and at *PDZK1* driven by an absence of effect in women (Fig. 2 and Table 2). For *ABCG2*, compared with women without the effect allele (referent group), the OR was 1.62 (95% CI 1.35–1.94) in women with the effect allele, 11.99 (10.81–13.30) in men without the effect allele, and 28.65 (25.73–31.90) in men with the effect allele (interaction *P* = 4.59 × 10^−5^). For *PDZK1*, compared with women without the effect allele (referent group), the OR was 0.92 (95% CI 0.77–1.10) in women with the effect allele, 10.54 (9.00–12.34) in men without the effect allele, and 13.61 (11.68–15.85) in men with the effect allele (interaction *P* = 3.67 × 10^−4^). For the other SNPs tested, no significant gene-sex interactions were observed (Table 2).

**Table 2:** Association and interaction between serum urate-associated single nucleotide polymorphisms and sex for gout risk

Gene	SNP	Effect allele	Women *n* = 188,221		Men *n* = 171,655		Gene-sex interaction *P*
			Effect allele absent Referent OR	Effect allele present OR (95% CI)	Effect allele absent OR (95% CI)	Effect allele present OR (95% CI)	
Loci replicated by Kottgen							
*ABCG2*	rs2231142	T	1	1.62 (1.35–1.94)	11.99 (10.81–13.30)	28.65 (25.73–31.90)	4.59 × 10^−5^
*SLC2A9*	rs12498742	A	1	4.12 (2.05–8.30)	18.09 (8.85–36.98)	55.62 (27.77–111.37)	0.42
*GCKR*	rs1260326	T	1	1.42 (1.18–1.70)	13.80 (11.77–16.18)	19.08 (16.33–22.29)	0.80
*SLC17A3*	rs1165151	T	1	0.76 (0.64–0.91)	13.15 (11.38–15.20)	10.56 (9.16–12.17)	0.60
*SLC22A12*	rs478607	A	1	0.78 (0.48–1.28)	13.96 (8.44–23.09)	10.57 (6.53–17.11)	0.90
*PDZK1*	rs1471633	A	1	0.92 (0.77–1.10)	10.54 (9.00–12.34)	13.61 (11.68–15.85)	3.67 × 10^−4^
*INHBE*	rs3741414	T	1	0.82 (0.69–0.98)	13.57 (12.16–15.15)	11.06 (9.89–12.38)	0.92
*SLC16A9*	rs1171614	T	1	0.95 (0.80–1.12)	14.28 (12.78–15.96)	11.74 (10.48–13.16)	0.11
*SLC22A11*	rs2078267	T	1	0.78 (0.65–0.95)	13.59 (11.42–16.17)	10.57 (8.93–12.51)	0.96
*RREB1*	rs675209	T	1	1.23 (1.04–1.45)	14.28 (12.62–16.16)	15.74 (13.90–17.82)	0.23
Loci reported by Kottgen							
*PKLR*	rs11264341	T	1	0.82 (0.69–0.97)	13.09 (11.4–15.08)	11.34 (9.87–13.02)	0.57
*INHBB*	rs17050272	A	1	1.10 (0.93–1.32)	14.25 (12.26–16.57)	14.54 (12.54–16.85)	0.40
*ACVR2A*	rs2307394	T	1	0.99 (0.75–1.31)	14.72 (11.13–19.48)	13.28 (10.14–17.40)	0.53
*MUSTN1*	rs6770152	T	1	0.96 (0.78–1.19)	15.29 (12.50–18.70)	12.75 (10.49–15.51)	0.21
*TMEM171*	rs17632159	C	1	0.76 (0.65–0.90)	12.81 (11.39–14.41)	11.31 (10.06–12.73)	0.11
*VEGFA*	rs729761	T	1	0.99 (0.84–1.17)	13.89 (12.29–15.70)	13.03 (11.53–14.74)	0.55
*MLXIPL*	rs1178977	A	1	1.71 (1.00–2.90)	17.96 (10.42–30.95)	22.86 (13.50–38.72)	0.30
*PRKAG2*	rs10480300	T	1	0.97 (0.82–1.15)	12.74 (11.31–14.35)	14.07 (12.50–15.85)	0.15
*STC1*	rs17786744	A	1	1.36 (1.06–1.74)	17.59 (13.86–22.33)	17.64 (13.99–22.25)	0.02
*HNF4G*	rs2941484	T	1	1.11 (0.92–1.33)	14.00 (11.89–16.49)	14.78 (12.59–17.34)	0.62
*ASAH2*	rs10821905	A	1	0.96 (0.80–1.15)	12.86 (11.57–14.29)	14.63 (13.12–16.33)	0.07
*LTBP3*	rs642803	T	1	0.92 (0.77–1.10)	13.81 (11.80–16.16)	12.33 (10.58–14.38)	0.76
*PTPN11*	rs653178	T	1	0.85 (0.70–1.02)	13.30 (11.24–15.74)	11.52 (9.78–13.57)	0.82
*NRG4*	rs1394125	A	1	1.02 (0.86–1.21)	13.06 (11.40–14.98)	14.17 (12.38–16.21)	0.52
*IGF1R*	rs6598541	A	1	1.06 (0.89–1.25)	13.05 (11.38–14.96)	14.48 (12.65–16.56)	0.59
*NFAT5*	rs7193778	T	1	1.12 (0.63–1.98)	20.20 (11.23–36.35)	14.91 (8.44–26.35)	0.17
*MAF*	rs7188445	A	1	0.96 (0.82–1.14)	13.64 (12.01–15.48)	12.91 (11.38–14.65)	0.84
*HLF*	rs7224610	A	1	0.90 (0.72–1.12)	13.76 (11.13–17.00)	12.13 (9.89–14.89)	0.88
*C17ORF82*	rs2079742	T	1	0.92 (0.52–1.64)	10.34 (5.65–18.93)	12.54 (7.09–22.19)	0.38
*PRPSAP1*	rs164009	A	1	1.32 (1.02–1.71)	17.00 (13.25–21.82)	17.27 (13.55–22.01)	0.05

Association and interaction data are reported according to effect allele presence or absence

Data are adjusted by age, body mass index, diuretic use, and renal failure

Experiment-wide significance is defined as *P* < 0.0017

*CI* confidence interval, *OR* odds ratio, *SNP* single nucleotide polymorphism

**Fig. 3:**
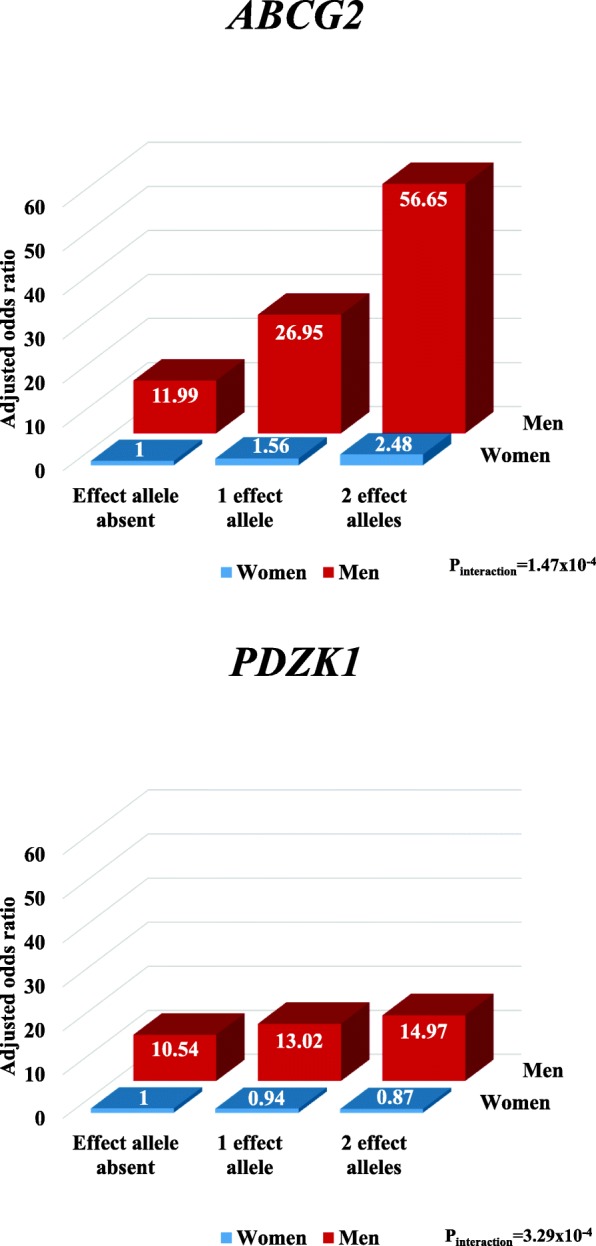
Association and interaction between serum urate-associated genetic variants (for *ABCG2* and *PDZK1*) and sex for gout risk according to the number of effect alleles present. Data are adjusted for age, body mass index, renal failure, and diuretic use. Experiment-wide significance is defined as *P* < 0.0017

Similar findings were found in the sensitivity analysis when excluding pre-menopausal women (Additional file 1: Table S4 and Additional file 2: Figure S1). When analysing for gene-sex interaction according to the number of effect alleles present, interaction was also observed for *ABCG2* and *PDZK1* in a similar pattern to that observed in the primary analysis (Fig. 3 and Additional file 1: Table S5).

## Discussion

In this large population of European ancestry, we have identified gene-sex interactions for *ABCG2* (rs2231142) and *PDZK1* (rs1471633) for gout risk, with the serum urate-associated SNPs exerting a greater influence on gout risk in men than in women. Consistent with prior reports [1–3], women with gout were older, had a higher body mass index, higher diuretic use, and more renal failure compared with men with gout. Importantly, all analyses examining genetic associations included age, body mass index, diuretic use, and renal failure within the regression models.

Sex-specific differences for *ABCG2* variants on serum urate have been previously reported. A GWAS by Dehghan et al. [16] which included a total of 26,714 participants across the Framingham cohort study, the Rotterdam cohort study, and the Atherosclerosis Risk in Communities (ARIC) study demonstrated significant *ABCG2* (rs2231142) gene-sex interactions for serum urate in participants of the Framingham study (of which almost all were of European descent) and for participants of European ancestry from the ARIC study. These differences were not observed in the Rotterdam cohort study or in African-American participants from the ARIC study. A 2009 meta-analysis by Kolz et al. [13] included 28,141 Europeans, and demonstrated that two *ABCG2* variants (rs2231142 and rs2199936) had significant effects on raising serum urate with the effect almost twice as strong in men compared with women. Differences for two *PDZK1* variants (rs12129861 and rs1471633) were also noted with urate-altering effects higher in men compared with women; however, the differences were not significant. Kottgen et al. [10] demonstrated similar differences in their large GWAS of > 140,000 Europeans for the rs2231142 variant with a serum urate raising effect of 0.270 mg/dl in men vs 0.181 mg/dl in women.

For gout risk, sex-specific differences for *ABCG2* variants have also been described. The Population Architecture using Genomics and Epidemiology (PAGE) study, which examined the association between gout and the *ABCG2* rs2231142 SNP, found a higher risk for gout in men than women in a population which included 13,783 European Americans, 4271 African Americans, and 1373 Mexican Americans [19]. In the Dehghan et al. [16] meta-analysis, sex-specific differences for gout risk were also noted for participants of European ancestry from the ARIC cohort with the rs2231142 variant exerting a greater risk of gout in men compared with women (OR 2.03 (95% CI 1.61–2.56) vs 1.07 (0.72–1.57), interaction *P* = 0.004). Contrasting results were found in a European and Eastern Polynesian population from New Zealand where gene-sex interactions for gout risk were not evident [20].

A causal mechanism for sex-specific differences with the *ABCG2* variant are unclear. There is strong evidence that oestrogen and progesterone reduce the risk of hyperuricaemia and development of gout due to their uricosuric effects [21–24]. Several studies in cell lines and animal models have shown that these hormones can regulate the activity of the ABCG2 transporter protein [25–28] and that these hormones may play a key role in ABCG2 transporter-mediated urate excretion at the level of the gut or kidney.

We are the first to report significant sex-specific differences for the *PDZK1* variant (rs1471633) on gout risk. The PDZK1 protein is not directly involved in urate transport but has been shown to be a key regulatory scaffolding protein in tethering other urate transporters (e.g. ABCG2, SLC22A11, and SLC17A1) to the multimolecular transportasome complex, and there is evidence that this complex may be responsible for controlling urate regulation at the level of the proximal renal tubule [29–31]. Studies reporting the association of *PDZK1* variants with gout have demonstrated mixed findings. Phipps-Green et al. [15] demonstrated an association between a *PDZK1* variant (rs1967017) and gout in a New Zealand European and Polynesian sample set with the effect allele exerting an increased risk of gout (OR 1.12 (95% CI 1.02–1.23)). The rs1967017 variant is the likely causal variant, with the urate-increasing allele causing increased *PDZK1* expression [32]. An association between the rs12129861 variant and gout was also found in a Japanese population (OR 0.80 (95% CI 0.67–0.96)) [33]. Similar findings have also been seen in a male Han Chinese population for both rs1967017 and rs12129861 *PDKZ1* variants [34]; however, this was not replicated for rs12129861 in a larger case-control study involving Han Chinese individuals [35]. These contrasting results may be attributed to different study populations, sample sizes, or differences in the number of men and women in the sample sets.

For the other SNPs tested, gene-sex interactions were not identified. This includes *SLC2A9* for which significant sex-specific differences for serum urate have been previously reported [10, 11, 13, 16]. In our study, *SLC2A9* had a large effect on gout risk in both men and women. However, we did not observe differential sex-specific differences for *SLC2A9* on gout risk in the interaction analysis. This may be because a high proportion (> 90%) of participants in the analysis had at least one *SLC2A9* effect allele and, in particular, there were very few women with gout who did not carry an effect allele (*n* = 8). This may have affected the power to detect sex-specific differences in gout risk for the *SLC2A9* variant. However, our findings are consistent with Dehghan et al. [16] who reported no evidence of a *SLC2A9* gene-sex interaction for gout risk despite reporting a significant differential sex-specific effect for serum urate.

Consistent with previous reports, *SLC2A9* and *ABCG2* variants exerted the highest risk for gout among the whole group [10, 15, 16]. Unlike these reports, our study shows that the *SLC2A9* variant exerts a greater risk of gout compared with the *ABCG2* variant. When calculating gout risk based on allelic odds ratios, however, this increased risk between the two variants is reversed with the *ABCG2* variant demonstrating an increased risk of gout compared with *SLC2A9* (Additional file 1: Table S2).

We acknowledge the limitations of this study. Firstly, our analysis was restricted to participants of European ancestry and our results may not be generalizable to populations of non-European ancestry. The age range for recruitment into the UK Biobank means that younger people with early onset gout, and older participants over the age of 70 years were not included in the analysis. Despite the large size of the UK Biobank, the number of women with gout in our analysis was low, which may have affected the power to detect small differences between groups. Co-morbidity and medication use data collected via the UK Biobank resource was through self-report. This method of data collection may not accurately represent the true prevalence of co-morbidities such as renal failure and medication use. However, this imprecision is likely to have applied systemically to all groups in the analysis. An assessment of sex-specific differences in serum urate would strengthen the findings of our study. However, serum urate measurements are not currently available in the UK Biobank database. Strengths of this study include the large sample size with consistent methods of data collection, and comprehensive assessment including patient interviews, hospitalisation records, and medical information.

## Conclusions

In people of European ancestry, gene-sex interactions for gout risk exist for *ABCG2* and *PDZK1*, with the effect alleles exerting a greater influence on gout risk in men than in women. In contrast, other serum urate-associated variants, including *SLC2A9*, do not demonstrate gene-sex interactions for gout risk.

## Additional files

Additional file 1: **Table S1.** Genotype frequencies of 30 serum urate-associated single-nucleotide polymorphisms according to group overall and sex. **Table S2.** Frequencies and association analysis of 30 serum urate-associated single-nucleotide polymorphisms for gout in all patients. **Table S3.** Frequencies and association analysis of 30 serum urate-associated single-nucleotide polymorphisms for gout. **Table S4.** Association and interaction between serum urate-associated single nucleotide polymorphisms and sex for gout risk, excluding pre-menopausal women. **Table S5.** Association and interaction between serum urate-associated single nucleotide polymorphisms and sex for gout risk according to the number of effect alleles. (DOCX 62 kb)

Additional file 2: **Figure S1.** Association and interaction between serum urate-associated genetic variants (for *ABCG2* and *PDZK1*) and sex for gout risk according to effect allele presence, excluding pre-menopausal women. Data are adjusted for age, body mass index, renal failure, and diuretic use. Experiment-wide significance is defined as *P* < 0.0017. (PPTX 44 kb)

## Abbreviations

*ABCG2*: ATP-binding cassette subfamily G member 2
ARIC: Atherosclerosis risk in communities
CI: Confidence interval
*GCKR*: Glucokinase regulator
GWAS: Genome-wide association study
*MLXIPL*: MAX-like protein X interacting protein-like
OR: Odds ratio
PAGE: Population architecture using genomics and epidemiology
*PDZK1*: PDZ domain containing 1
SD: Standard deviation
*SLC17A1*: Solute carrier family 17 member 1
*SLC22A11*: Solute carrier family 22 member 11
*SLC2A9*: Solute carrier family 2 member 9
SNP: Single nucleotide polymorphism
