# Supplementary material for: Interactions between serum urate-associated genetic variants and sex on gout risk: analysis of the UK Biobank
Source: Arthritis Res Ther. 2019 Jan 9;21:13. doi: 10.1186/s13075-018-1787-5 (PMC6327586; doi:10.1186/s13075-018-1787-5)
Supplement: Supplementary file 1 — Table S1. Genotype frequencies of 30 serum urate-associated single-nucleotide polymorphisms according to group overall and sex. Table S2. Frequencies and association analysis of 30 serum urate-associated single-nucleotide polymorphisms for gout in all patients. Table S3. Frequencies and association analysis of 30 serum urate-associated single-nucleotide polymorphisms for gout. Table S4. Association and interaction between serum urate-associated single nucleotide polymorphisms and sex for gout risk, excluding pre-menopausal women. Table S5. Association and interaction between serum urate-associated single nucleotide polymorphisms and sex for gout risk according to the number of effect alleles. (DOCX 62 kb) [file 13075_2018_1787_MOESM1_ESM.docx]

**Additional file 1**

**Table S1:** **Genotype frequencies of 30 serum urate-associated single-nucleotide polymorphisms according to group overall and sex**

| **Gene** | **SNP** | **A1** | **A2** | **Genotype** | **All patients**  **n=359876** | | **Women**  **n=188221** | | **Men**  **n=171655** | |
| --- | --- | --- | --- | --- | --- | --- | --- | --- | --- | --- |
|  |  |  |  |  | **Control**  **n=352534**  **n (%)** | **Gout**  **n=7342**  **n (%)** | **Control**  **n=187647**  **n (%)** | **Gout**  **n=574**  **n (%)** | **Control**  **n=164887**  **n (%)** | **Gout**  **n=6768**  **n (%)** |
| **Loci replicated by Köttgen** | | | | | | | | | | |
| ***ABCG2*** | rs2231142 | T | G | GG | 278344 (79.0%) | 4644 (63.3%) | 147672 (78.7%) | 401 (69.9%) | 130672 (79.2%) | 4243 (62.7%) |
|  |  |  |  | TG | 69684 (19.8%) | 2397 (32.6%) | 37517 (20.0%) | 157 (27.4%) | 32167 (19.5%) | 2240 (33.1%) |
|  |  |  |  | TT | 4506 (1.3%) | 301 (4.1%) | 2458 (1.3%) | 16 (2.8%) | 2048 (1.2%) | 285 (4.2%) |
| ***SLC2A9*** | rs12498742 | A | G | AA | 206217 (58.5%) | 5284 (72.0%) | 110098 (58.7%) | 444 (77.4%) | 96119 (58.3%) | 4840 (71.5%) |
|  |  |  |  | GA | 126477 (35.9%) | 1915 (26.1%) | 67111 (35.8%) | 122 (21.3%) | 59366 (36.0%) | 1793 (26.5%) |
|  |  |  |  | GG | 19467 (5.5%) | 140 (1.9%) | 10235 (5.5%) | 8 (1.4%) | 9232 (5.6%) | 132 (2.0%) |
| ***GCKR*** | rs1260326 | T | C | CC | 129371 (36.7%) | 2209 (30.1%) | 68937 (36.7%) | 167 (29.1%) | 60434 (36.7%) | 2042 (30.2%) |
|  |  |  |  | TC | 168152 (47.7%) | 3693 (50.3%) | 89498 (47.7%) | 293 (51.0%) | 78654 (47.7%) | 3400 (50.2%) |
|  |  |  |  | TT | 55011 (15.6%) | 1440 (19.6%) | 29212 (15.6%) | 114 (19.9%) | 25799 (15.6%) | 1326 (19.6%) |
| ***SLC17A3*** | rs1165151 | T | G | GG | 105370 (29.9%) | 2525 (34.4%) | 56240 (30.0%) | 206 (35.9%) | 49130 (29.8%) | 2319 (34.3%) |
|  |  |  |  | TG | 174509 (49.5%) | 3575 (48.7%) | 92954 (49.5%) | 262 (45.6%) | 81555 (49.5%) | 3313 (49.0%) |
|  |  |  |  | TT | 72161 (20.5%) | 1229 (16.7%) | 38198 (20.4%) | 104 (18.1%) | 33963 (20.6%) | 1125 (16.6%) |
| ***SLC22A12*** | rs478607 | A | G | AA | 253523 (71.9%) | 5068 (69.0%) | 134663 (71.8%) | 402 (70.0%) | 118860 (72.1%) | 4666 (68.9%) |
|  |  |  |  | GA | 89852 (25.5%) | 2049 (27.9%) | 48044 (25.6%) | 155 (27.0%) | 41808 (25.4%) | 1894 (28.0%) |
|  |  |  |  | GG | 8074 (2.3%) | 210 (2.9%) | 4390 (2.3%) | 17 (3.0%) | 3684 (2.2%) | 193 (2.9%) |
| ***PDZK1*** | rs1471633 | A | C | AA | 75897 (21.5%) | 1820 (24.8%) | 40512 (21.6%) | 115 (20.0%) | 35385 (21.5%) | 1705 (25.2%) |
|  |  |  |  | AC | 174960 (49.6%) | 3725 (50.7%) | 92961 (49.5%) | 285 (49.7%) | 81999 (49.7%) | 3440 (50.8%) |
|  |  |  |  | CC | 101351 (28.7%) | 1791 (24.4%) | 54012 (28.8%) | 174 (30.3%) | 47339 (28.7%) | 1617 (23.9%) |
| ***INHBE*** | rs3741414 | T | C | CC | 202597 (57.5%) | 4550 (62.0%) | 107929 (57.5%) | 354 (61.7%) | 94668 (57.4%) | 4196 (62.0%) |
|  |  |  |  | TC | 128845 (36.5%) | 2489 (33.9%) | 68459 (36.5%) | 193 (33.6%) | 60386 (36.6%) | 2296 (33.9%) |
|  |  |  |  | TT | 20879 (5.9%) | 299 (4.1%) | 11151(5.9%) | 27 (4.7%) | 9728 (5.9%) | 272 (4.0%) |
| ***SLC16A9*** | rs1171614 | T | C | CC | 207797 (58.9%) | 4646 (63.3%) | 110691 (59.0%) | 345 (60.1%) | 97106 (58.9%) | 4301 (63.5%) |
|  |  |  |  | TC | 124600 (35.3%) | 2397 (32.6%) | 66213 (35.3%) | 203 (35.4%) | 58387 (35.4%) | 2194 (32.4%) |
|  |  |  |  | TT | 18658 (5.3%) | 276 (3.8%) | 9938 (5.3%) | 24 (4.2%) | 8720 (5.3%) | 252 (3.7%) |
| ***SLC22A11*** | rs2078267 | T | C | CC | 72041 (20.4%) | 1803 (24.6%) | 38615 (20.6%) | 141 (24.6%) | 33426 (20.3%) | 1662 (24.6%) |
|  |  |  |  | CT | 174736 (49.6%) | 3671 (50.0%) | 93212 (49.7%) | 270 (47.0%) | 81524 (49.4%) | 3401 (50.3%) |
|  |  |  |  | TT | 105757 (30.0%) | 1868 (25.4%) | 55820 (29.7%) | 163 (28.4%) | 49937 (30.3%) | 1705 (25.2%) |
| ***RREB1*** | rs675209 | T | C | CC | 188347 (53.4%) | 3746 (51.0%) | 100088 (53.3%) | 277 (48.3%) | 88259 (53.5%) | 3469 (51.3%) |
|  |  |  |  | TC | 138408 (39.3%) | 3015 (41.1%) | 73791 (39.3%) | 248 (43.2%) | 64617 (39.2%) | 2767 (40.9%) |
|  |  |  |  | TT | 25779 (7.3%) | 581 (7.9%) | 13768 (7.3%) | 49 (8.5%) | 12011 (7.3%) | 532 (7.9%) |
| **Loci reported by Köttgen** | | | | | | | | | | |
| ***PKLR*** | rs11264341 | T | C | CC | 115516 (32.8%) | 2655 (36.2%) | 61277 (32.7%) | 216 (37.6%) | 54239 (32.9%) | 2439 (36.0%) |
|  |  |  |  | TC | 172147 (48.8%) | 3510 (47.8%) | 91760 (48.9%) | 265 (46.2%) | 80387 (48.8%) | 3245 (47.9%) |
|  |  |  |  | TT | 64871 (18.4%) | 1177 (16.0%) | 34610 (18.4%) | 93 (16.2%) | 30261 (18.4%) | 1084 (16.0%) |
| ***INHBB*** | rs17050272 | A | G | AA | 59141 (16.8%) | 1306 (17.8%) | 31416 (16.7%) | 98 (17.1%) | 27725 (16.8%) | 1208 (17.8%) |
|  |  |  |  | AG | 170276 (48.3%) | 3517 (47.9%) | 90691 (48.3%) | 288 (50.2%) | 79585 (48.3%) | 3229 (47.7%) |
|  |  |  |  | GG | 123117 (34.9%) | 2519 (34.3%) | 65540 (34.9%) | 188 (32.8%) | 57577 (34.9%) | 2331 (34.4%) |
| ***ACVR2A*** | rs2307394 | T | C | CC | 32267 (9.2%) | 734 (10.0%) | 17337 (9.2%) | 55 (9.6%) | 14930 (9.1%) | 679 (10.0%) |
|  |  |  |  | CT | 149683 (42.5%) | 3106 (42.3%) | 79765 (42.5%) | 259 (45.1%) | 69918 (42.4%) | 2847 (42.1%) |
|  |  |  |  | TT | 170584 (48.4%) | 3502 (47.7%) | 90545 (48.3%) | 260 (45.3%) | 80039 (48.5%) | 3242 (47.9%) |
| ***MUSTN1*** | rs6770152 | T | G | GG | 62256 (17.7%) | 1480 (20.2%) | 33137 (17.7%) | 105 (18.3%) | 29119 (17.7%) | 1375 (20.3%) |
|  |  |  |  | GT | 169816 (48.2%) | 3560 (48.5%) | 90375 (48.2%) | 290 (50.5%) | 79441 (48.2%) | 3270 (48.3%) |
|  |  |  |  | TT | 115502 (32.8%) | 2198 (29.9%) | 61452 (32.7%) | 167 (29.1%) | 54050 (32.8%) | 2031 (30.0%) |
| ***TMEM171*** | rs17632159 | C | G | CC | 32182 (9.1%) | 603 (8.2%) | 17121 (9.1%) | 51 (8.9%) | 15061 (9.1%) | 552 (8.2%) |
|  |  |  |  | CG | 147766 (41.9%) | 2922 (39.8%) | 78656 (41.9%) | 201 (35.0%) | 69110 (41.9%) | 2721 (40.2%) |
|  |  |  |  | GG | 169402 (48.1%) | 3763 (51.3%) | 90196 (48.1%) | 312 (54.4%) | 79206 (48.0%) | 3451 (51.0%) |
| ***VEGFA*** | rs729761 | T | G | GG | 174642 (49.5%) | 3725 (50.7%) | 93109 (49.6%) | 286 (49.8%) | 81533 (49.4%) | 3439 (50.8%) |
|  |  |  |  | TG | 137684 (39.1%) | 2803 (38.2%) | 73057 (38.9%) | 221 (38.5%) | 64627 (39.2%) | 2582 (38.2%) |
|  |  |  |  | TT | 27605 (7.8%) | 551 (7.5%) | 14646 (7.8%) | 46 (8.0%) | 12959 (7.9%) | 505 (7.5%) |
| ***MLXIPL*** | rs1178977 | A | G | AA | 227499 (64.5%) | 5040 (68.6%) | 121157 (64.6%) | 411 (71.6%) | 106342 (64.5%) | 4629 (68.4%) |
|  |  |  |  | GA | 111279 (31.6%) | 2075 (28.3%) | 59205 (31.6%) | 149 (26.0%) | 52074 (31.6%) | 1926 (28.5%) |
|  |  |  |  | GG | 13703 (3.9%) | 225 (3.1%) | 7258 (3.9%) | 14 (2.4%) | 6445 (3.9%) | 211 (3.1%) |
| ***PRKAG2*** | rs10480300 | T | C | CC | 184444 (52.3%) | 3666 (49.9%) | 98248 (52.4%) | 304 (53.0%) | 86196 (52.3%) | 3362 (49.7%) |
|  |  |  |  | TC | 138214 (39.2%) | 2943 (40.1%) | 73479 (39.2%) | 201 (35.0%) | 64735 (39.3%) | 2742 (40.5%) |
|  |  |  |  | TT | 26432 (7.5%) | 648 (8.8%) | 14063 (7.5%) | 61 (10.6%) | 12369 (7.5%) | 587 (8.7%) |
| ***STC1*** | rs17786744 | A | G | AA | 121792 (34.5%) | 2457 (33.5%) | 64841 (34.6%) | 221 (38.5%) | 56951 (34.5%) | 2236 (33.0%) |
|  |  |  |  | GA | 167919 (47.6%) | 3575 (48.7%) | 89328 (47.6%) | 270 (47.0%) | 78591 (47.7%) | 3305 (48.8%) |
|  |  |  |  | GG | 58492 (16.6%) | 1201 (16.4%) | 31198 (16.6%) | 73 (12.7%) | 27294 (16.6%) | 1128 (16.7%) |
| ***HNF4G*** | rs2941484 | T | C | CC | 104945 (29.8%) | 2113 (28.8%) | 55832 (29.8%) | 158 (27.5%) | 49113 (29.8%) | 1955 (28.9%) |
|  |  |  |  | TC | 168129 (47.7%) | 3471 (47.3%) | 89507 (47.7%) | 277 (48.3%) | 78622 (47.7%) | 3194 (47.2%) |
|  |  |  |  | TT | 68142 (19.3%) | 1521 (20.7%) | 36384 (19.4%) | 121 (21.1%) | 31758 (19.3%) | 1400 (20.7%) |
| ***ASAH2*** | rs10821905 | A | G | AA | 10944 (3.1%) | 282 (3.8%) | 5837 (3.1%) | 21 (3.7%) | 5107 (3.1%) | 261 (3.9%) |
|  |  |  |  | AG | 101393 (28.8%) | 2230 (30.4%) | 54072 (28.8%) | 155 (27.0%) | 47321 (28.7%) | 2075 (30.7%) |
|  |  |  |  | GG | 235590 (66.8%) | 4720 (64.3%) | 125266 (66.8%) | 386 (67.2%) | 110324 (66.9%) | 4334 (64.0%) |
| ***LTBP3*** | rs642803 | T | C | CC | 99696 (28.3%) | 2245 (30.6%) | 53091 (28.3%) | 173 (30.1%) | 46605 (28.3%) | 2072 (30.6%) |
|  |  |  |  | TC | 172770 (49.0%) | 3603 (49.1%) | 91715 (48.9%) | 277 (48.3%) | 81055 (49.2%) | 3326 (49.1%) |
|  |  |  |  | TT | 75868 (21.5%) | 1405 (19.1%) | 40584 (21.6%) | 117 (20.4%) | 35284 (21.4%) | 1288 (19.0%) |
| ***PTPN11*** | rs653178 | T | C | CC | 82736 (23.5%) | 1900 (25.9%) | 44185 (23.5%) | 150 (26.1%) | 38551 (23.4%) | 1750 (25.9%) |
|  |  |  |  | CT | 175842 (49.9%) | 3589 (48.9%) | 93483 (49.8%) | 276 (48.1%) | 82359 (49.9%) | 3313 (49.0%) |
|  |  |  |  | TT | 93956 (26.7%) | 1853 (25.2%) | 49979 (26.6%) | 148 (25.8%) | 43977 (26.7%) | 1705 (25.2%) |
| ***NRG4*** | rs1394125 | A | G | AA | 46133 (13.1%) | 1067 (14.5%) | 24551 (13.1%) | 83 (14.5%) | 21582 (13.1%) | 984 (14.5%) |
|  |  |  |  | AG | 163097 (46.3%) | 3420 (46.6%) | 87057 (46.4%) | 263 (45.8%) | 76040 (46.1%) | 3157 (46.6%) |
|  |  |  |  | GG | 143304 (40.6%) | 2855 (38.9%) | 76039 (40.5%) | 228 (39.7%) | 67265 (40.8%) | 2627 (38.8%) |
| ***IGF1R*** | rs6598541 | A | G | AA | 43863 (12.4%) | 971 (13.2%) | 23187 (12.4%) | 82 (14.3%) | 20676 (12.5%) | 889 (13.1%) |
|  |  |  |  | AG | 159753 (45.3%) | 3449 (47.0%) | 85237 (45.4%) | 262 (45.6%) | 74516 (45.2%) | 3187 (47.1%) |
|  |  |  |  | GG | 145191 (41.2%) | 2850 (38.8%) | 77196 (41.1%) | 228 (39.7%) | 67995 (41.2%) | 2622 (38.7%) |
| ***NFAT5*** | rs7193778 | T | C | CC | 7688 (2.2%) | 209 (2.8%) | 4140 (2.2%) | 12 (2.1%) | 3548 (2.2%) | 197 (2.9%) |
|  |  |  |  | CT | 88526 (25.1%) | 2011 (27.4%) | 47121 (25.1%) | 156 (27.2%) | 41405 (25.1%) | 1855 (27.4%) |
|  |  |  |  | TT | 254430 (72.2%) | 5084 (69.2%) | 135401 (72.2%) | 404 (70.4%) | 119029 (72.2%) | 4680 (69.1%) |
| ***MAF*** | rs7188445 | A | G | AA | 37524 (10.6%) | 770 (10.5%) | 19802 (10.6%) | 47 (8.2%) | 17722 (10.7%) | 723 (10.7%) |
|  |  |  |  | AG | 153716 (43.6%) | 3136 (42.7%) | 81809 (43.6%) | 258 (44.9%) | 71907 (43.6%) | 2878 (42.5%) |
|  |  |  |  | GG | 158842 (45.1%) | 3394 (46.2%) | 84669 (45.1%) | 266 (46.3%) | 74173 (45.0%) | 3128 (46.2%) |
| ***HLF*** | rs7224610 | A | C | AA | 123132 (34.9%) | 2415 (32.9%) | 65433 (34.9%) | 186 (32.4%) | 57699 (35.0%) | 2229 (32.9%) |
|  |  |  |  | CA | 160860 (45.6%) | 3389 (46.2%) | 85676 (45.7%) | 268 (46.7%) | 75184 (45.6%) | 3121 (46.1%) |
|  |  |  |  | CC | 53397 (15.1%) | 1226 (16.7%) | 28403 (15.1%) | 95 (16.6%) | 24994 (15.2%) | 1131 (16.7%) |
| ***C17ORF82*** | rs2079742 | T | C | CC | 6466 (1.8%) | 116 (1.6%) | 3459 (1.8%) | 12 (2.1%) | 3007 (1.8%) | 104 (1.5%) |
|  |  |  |  | CT | 81704 (23.2%) | 1618 (22.0%) | 43896 (23.4%) | 132 (23.0%) | 37808 (22.9%) | 1486 (22.0%) |
|  |  |  |  | TT | 256342 (72.7%) | 5440 (74.1%) | 135984 (72.5%) | 416 (72.5%) | 120358 (73.0%) | 5024 (74.2%) |
| ***PRPSAP1*** | rs164009 | A | G | AA | 129913 (36.9%) | 2779 (37.9%) | 69353 (37.0%) | 215 (37.5%) | 60560 (36.7%) | 2564 (37.9%) |
|  |  |  |  | GA | 165823 (47.0%) | 3404 (46.4%) | 88219 (47.0%) | 286 (49.8%) | 77604 (47.1%) | 3118 (46.1%) |
|  |  |  |  | GG | 52963 (15.0%) | 1084 (14.8%) | 28012 (14.9%) | 67 (11.7%) | 24951 (15.1%) | 1017 (15.0%) |

SNP, single nucleotide polymorphism.

**Table S2:** **Frequencies and association analysis of 30 serum urate-associated single-nucleotide polymorphisms for gout in all patients**

| **Gene** | **SNP** | **Effect allele** | **All patients**  **n=359876** | | | | | |
| --- | --- | --- | --- | --- | --- | --- | --- | --- |
|  |  |  | **0 effect alleles** | | **1 effect allele** | | **2 effect alleles** | |
|  |  |  | **Freq (control)** | **Freq (gout)** | **Freq (control)** | **Freq (gout)** | **Freq (control)** | **Freq (gout)** |
|  |  |  | **Allelic OR for gout (95% CI), P** | | | | | |
| **Loci replicated by Köttgen** | | | | | | | | |
| ***ABCG2*** | rs2231142 | T | 0.79 | 0.63 | 0.20 | 0.33 | 0.01 | 0.04 |
|  |  |  | **2.11** (2.02-2.20), **1.03x10^-269^** | | | | | |
| ***SLC2A9*** | rs12498742 | A | 0.06 | 0.02 | 0.36 | 0.26 | 0.59 | 0.72 |
|  |  |  | **1.76** (1.68-1.84), **7.74x10^-127^** | | | | | |
| ***GCKR*** | rs1260326 | T | 0.37 | 0.30 | 0.48 | 0.50 | 0.16 | 0.20 |
|  |  |  | **1.26** (1.22-1.31), **1.20x10^-42^** | | | | | |
| ***SLC17A3*** | rs1165151 | T | 0.30 | 0.35 | 0.50 | 0.49 | 0.21 | 0.17 |
|  |  |  | **0.84** (0.81-0.87), **8.07x10^-24^** | | | | | |
| ***SLC22A12*** | rs478607 | A | 0.02 | 0.03 | 0.26 | 0.28 | 0.72 | 0.69 |
|  |  |  | **0.87** (0.83-0.91), **2.28x10^-10^** | | | | | |
| ***PDZK1*** | rs1471633 | A | 0.29 | 0.24 | 0.50 | 0.51 | 0.22 | 0.25 |
|  |  |  | **1.17** (1.13-1.20), **1.79x10^-19^** | | | | | |
| ***INHBE*** | rs3741414 | T | 0.58 | 0.62 | 0.37 | 0.34 | 0.06 | 0.04 |
|  |  |  | **0.82** (0.79-0.86), **7.73x10^-21^** | | | | | |
| ***SLC16A9*** | rs1171614 | T | 0.59 | 0.64 | 0.36 | 0.33 | 0.05 | 0.04 |
|  |  |  | **0.84** (0.81-0.88), **1.26x10^-16^** | | | | | |
| ***SLC22A11*** | rs2078267 | T | 0.20 | 0.25 | 0.50 | 0.50 | 0.30 | 0.25 |
|  |  |  | **0.83** (0.81-0.86), **3.86x10^-27^** | | | | | |
| ***RREB1*** | rs675209 | T | 0.53 | 0.51 | 0.39 | 0.41 | 0.07 | 0.08 |
|  |  |  | **1.08** (1.04-1.12), **2.92x10^-5^** | | | | | |
| **Loci reported by Köttgen** | | | | | | | | |
| ***PKLR*** | rs11264341 | T | 0.33 | 0.36 | 0.49 | 0.48 | 0.18 | 0.16 |
|  |  |  | **0.89** (0.86-0.92), **4.39x10^-11^** | | | | | |
| ***INHBB*** | rs17050272 | A | 0.35 | 0.34 | 0.48 | 0.48 | 0.17 | 0.18 |
|  |  |  | **1.03** (1.00-1.07), **0.08** | | | | | |
| ***ACVR2A*** | rs2307394 | T | 0.09 | 0.10 | 0.43 | 0.42 | 0.48 | 0.48 |
|  |  |  | **0.97** (0.94-1.00), **0.08** | | | | | |
| ***MUSTN1*** | rs6770152 | T | 0.18 | 0.20 | 0.49 | 0.49 | 0.33 | 0.30 |
|  |  |  | **0.90** (0.87-0.92), **1.12x10^-10^** | | | | | |
| ***TMEM171*** | rs17632159 | C | 0.49 | 0.51 | 0.42 | 0.40 | 0.09 | 0.08 |
|  |  |  | **0.90** (0.87-0.94), **4.66x10^-8^** | | | | | |
| ***VEGFA*** | rs729761 | T | 0.51 | 0.53 | 0.41 | 0.40 | 0.08 | 0.08 |
|  |  |  | **0.96** (0.92-0.99), **0.02** | | | | | |
| ***MLXIPL*** | rs1178977 | A | 0.04 | 0.03 | 0.32 | 0.28 | 0.65 | 0.69 |
|  |  |  | **1.20** (1.15-1.25), **1.01x10^-15^** | | | | | |
| ***PRKAG2*** | rs10480300 | T | 0.53 | 0.51 | 0.40 | 0.41 | 0.08 | 0.09 |
|  |  |  | **1.10** (1.06-1.14), **2.72x10^-7^** | | | | | |
| ***STC1*** | rs17786744 | A | 0.17 | 0.17 | 0.48 | 0.49 | 0.35 | 0.34 |
|  |  |  | **0.99** (0.96-1.02), **0.48** | | | | | |
| ***HNF4G*** | rs2941484 | T | 0.31 | 0.30 | 0.49 | 0.49 | 0.20 | 0.21 |
|  |  |  | **1.05** (1.07-1.09), **3.06x10^-3^** | | | | | |
| ***ASAH2*** | rs10821905 | A | 0.68 | 0.65 | 0.29 | 0.31 | 0.03 | 0.04 |
|  |  |  | **1.11** (1.07-1.16), **6.22x10^-7^** | | | | | |
| ***LTBP3*** | rs642803 | T | 0.29 | 0.31 | 0.50 | 0.50 | 0.22 | 0.19 |
|  |  |  | **0.91** (0.88-0.94), **3.77x10^-8^** | | | | | |
| ***PTPN11*** | rs653178 | T | 0.24 | 0.26 | 0.50 | 0.49 | 0.27 | 0.25 |
|  |  |  | **0.92** (0.89-0.95), **4.03x10^-7^** | | | | | |
| ***NRG4*** | rs1394125 | A | 0.41 | 0.39 | 0.46 | 0.47 | 0.13 | 0.15 |
|  |  |  | **1.07** (1.04-1.11), **4.94x10^-5^** | | | | | |
| ***IGF1R*** | rs6598541 | A | 0.42 | 0.39 | 0.46 | 0.47 | 0.13 | 0.13 |
|  |  |  | **1.07** (1.03-1.11), **1.79x10^-4^** | | | | | |
| ***NFAT5*** | rs7193778 | T | 0.02 | 0.03 | 0.25 | 0.28 | 0.73 | 0.70 |
|  |  |  | **0.89** (0.85-0.93), **1.03x10^-7^** | | | | | |
| ***MAF*** | rs7188445 | A | 0.45 | 0.47 | 0.44 | 0.43 | 0.11 | 0.11 |
|  |  |  | **0.97** (0.94-1.01), **0.09** | | | | | |
| ***HLF*** | rs7224610 | A | 0.16 | 0.17 | 0.48 | 0.48 | 0.37 | 0.34 |
|  |  |  | **0.92** (0.89-0.95), **2.62x10^-6^** | | | | | |
| ***C17ORF82*** | rs2079742 | T | 0.02 | 0.02 | 0.24 | 0.23 | 0.74 | 0.76 |
|  |  |  | **1.08** (1.03-1.13), **3.07x10^-3^** | | | | | |
| ***PRPSAP1*** | rs164009 | A | 0.12 | 0.15 | 0.48 | 0.47 | 0.37 | 0.38 |
|  |  |  | **1.03** (0.99-1.06), **0.13** | | | | | |
| ***Number of loci associated with gout at experiment-wide significance*** | | | **22** | | | | | |

Allelic odds ratios are used to report association data. CI, Confidence interval; Freq, frequency; SNP, single nucleotide polymorphism. Data are adjusted for age, body mass index, diuretic use and renal failure. Experiment wide-significance is defined as P < 0.0017.

**Table S3:** **Frequencies and association analysis of 30 serum urate-associated single-nucleotide polymorphisms for gout according to sex**

| **Gene** | **SNP** | **Effect allele** | **Women**  **n=188221** | | | | | | **Men**  **n=171655** | | | | | |
| --- | --- | --- | --- | --- | --- | --- | --- | --- | --- | --- | --- | --- | --- | --- |
|  |  |  | **0 effect alleles** | | **1 effect allele** | | **2 effect alleles** | | **0 effect alleles** | | **1 effect allele** | | **2 effect alleles** | |
|  |  |  | **Freq (control)** | **Freq (gout)** | **Freq (control)** | **Freq (gout)** | **Freq (control)** | **Freq (gout)** | **Freq (control)** | **Freq (gout)** | **Freq (control)** | **Freq (gout)** | **Freq (control)** | **Freq (gout)** |
|  |  |  | **Allelic OR for gout (95% CI), P** | | | | | | **Allelic OR for gout (95% CI), P** | | | | | |
| **Loci replicated by Köttgen** | | | | | | | | | | | | | | |
| ***ABCG2*** | rs2231142 | T | 0.79 | 0.70 | 0.20 | 0.27 | 0.01 | 0.03 | 0.79 | 0.63 | 0.20 | 0.33 | 0.01 | 0.04 |
|  |  |  | **1.58** (1.35-1.85), **1.74x10^-8^** | | | | | | **2.21** (2.12-2.31), **1.27x10^-269^** | | | | | |
| ***SLC2A9*** | rs12498742 | A | 0.06 | 0.01 | 0.36 | 0.21 | 0.59 | 0.77 | 0.06 | 0.02 | 0.36 | 0.27 | 0.58 | 0.72 |
|  |  |  | **2.23** (1.87-2.67), **1.55x10^-18^** | | | | | | **1.75** (1.66-1.83), **1.23x10^-112^** | | | | | |
| ***GCKR*** | rs1260326 | T | 0.37 | 0.29 | 0.48 | 0.51 | 0.16 | 0.20 | 0.37 | 0.30 | 0.48 | 0.50 | 0.16 | 0.20 |
|  |  |  | **1.28** (1.14-1.44), **3.43x10^-5^** | | | | | | **1.27** (1.22-1.31), **1.64x10^-39^** | | | | | |
| ***SLC17A3*** | rs1165151 | T | 0.30 | 0.36 | 0.50 | 0.46 | 0.20 | 0.18 | 0.30 | 0.34 | 0.50 | 0.49 | 0.21 | 0.17 |
|  |  |  | **0.84** (0.75-0.95), **0.01** | | | | | | **0.84** (0.81-0.87), **8.24x10^-23^** | | | | | |
| ***SLC22A12*** | rs478607 | A | 0.02 | 0.03 | 0.26 | 0.27 | 0.72 | 0.70 | 0.02 | 0.03 | 0.25 | 0.28 | 0.72 | 0.69 |
|  |  |  | **0.91** (0.78-1.06), **0.23** | | | | | | **0.86** (0.82-0.90), **2.02x10^-10^** | | | | | |
| ***PDZK1*** | rs1471633 | A | 0.28 | 0.30 | 0.50 | 0.50 | 0.22 | 0.20 | 0.29 | 0.24 | 0.50 | 0.51 | 0.22 | 0.25 |
|  |  |  | **0.93** (0.83-1.05), **0.24** | | | | | | **1.19** (1.15-1.23), **1.85x10^-22^** | | | | | |
| ***INHBE*** | rs3741414 | T | 0.58 | 0.62 | 0.37 | 0.34 | 0.06 | 0.05 | 0.58 | 0.62 | 0.37 | 0.34 | 0.06 | 0.04 |
|  |  |  | **0.84** (0.73-0.97), **0.02** | | | | | | **0.82** (0.79-0.86), **1.67x10^-19^** | | | | | |
| ***SLC16A9*** | rs1171614 | T | 0.59 | 0.60 | 0.35 | 0.36 | 0.05 | 0.04 | 0.59 | 0.64 | 0.36 | 0.33 | 0.05 | 0.04 |
|  |  |  | **0.94** (0.82-1.09), **0.41** | | | | | | **0.83** (0.80-0.87), **1.01x10^-16^** | | | | | |
| ***SLC22A11*** | rs2078267 | T | 0.21 | 0.25 | 0.50 | 0.47 | 0.30 | 0.28 | 0.20 | 0.25 | 0.49 | 0.50 | 0.30 | 0.25 |
|  |  |  | **0.89** (0.79-1.00), **0.05** | | | | | | **0.82** (0.80-0.85), **1.77x10^-27^** | | | | | |
| ***RREB1*** | rs675209 | T | 0.53 | 0.48 | 0.39 | 0.43 | 0.07 | 0.09 | 0.54 | 0.51 | 0.39 | 0.41 | 0.07 | 0.08 |
|  |  |  | **1.16** (1.02-1.32), **0.02** | | | | | | **1.08** (1.04-1.12), **1.15x10^-4^** | | | | | |
| **Loci reported by Köttgen** | | | | | | | | | | | | | | |
| ***PKLR*** | rs11264341 | T | 0.33 | 0.38 | 0.49 | 0.46 | 0.18 | 0.16 | 0.33 | 0.36 | 0.49 | 0.48 | 0.18 | 0.16 |
|  |  |  | **0.88** (0.78-1.00), **0.04** | | | | | | **0.89** (0.86-0.92), **2.27x10^-10^** | | | | | |
| ***INHBB*** | rs17050272 | A | 0.35 | 0.33 | 0.48 | 0.50 | 0.17 | 0.17 | 0.35 | 0.34 | 0.48 | 0.48 | 0.17 | 0.18 |
|  |  |  | **1.04** (0.93-1.18), **0.47** | | | | | | **1.03** (1.00-1.07), **0.09** | | | | | |
| ***ACVR2A*** | rs2307394 | T | 0.09 | 0.10 | 0.43 | 0.45 | 0.48 | 0.45 | 0.09 | 0.10 | 0.42 | 0.42 | 0.49 | 0.48 |
|  |  |  | **0.94** (0.83-1.06), **0.30** | | | | | | **0.97** (0.93-1.01), **0.09** | | | | | |
| ***MUSTN1*** | rs6770152 | T | 0.18 | 0.19 | 0.49 | 0.52 | 0.33 | 0.30 | 0.18 | 0.21 | 0.49 | 0.49 | 0.33 | 0.30 |
|  |  |  | **0.92** (0.82-1.04), **0.17** | | | | | | **0.89** (0.86-0.92), **2.73x10^-10^** | | | | | |
| ***TMEM171*** | rs17632159 | C | 0.49 | 0.55 | 0.42 | 0.36 | 0.09 | 0.09 | 0.49 | 0.51 | 0.42 | 0.41 | 0.09 | 0.08 |
|  |  |  | **0.85** (0.75-0.97), **0.02** | | | | | | **0.90** (0.87-0.94), **2.36x10^-7^** | | | | | |
| ***VEGFA*** | rs729761 | T | 0.52 | 0.52 | 0.40 | 0.40 | 0.08 | 0.08 | 0.51 | 0.53 | 0.41 | 0.40 | 0.08 | 0.08 |
|  |  |  | **1.01** (0.88-1.15), **0.89** | | | | | | **0.95** (0.91-0.99), **0.01** | | | | | |
| ***MLXIPL*** | rs1178977 | A | 0.04 | 0.02 | 0.32 | 0.26 | 0.65 | 0.72 | 0.04 | 0.03 | 0.32 | 0.29 | 0.65 | 0.68 |
|  |  |  | **1.37** (1.17-1.61), **1.35x10^-4^** | | | | | | **1.19** (1.13-1.24), **3.18x10^-13^** | | | | | |
| ***PRKAG2*** | rs10480300 | T | 0.53 | 0.54 | 0.40 | 0.36 | 0.08 | 0.11 | 0.53 | 0.50 | 0.40 | 0.41 | 0.08 | 0.09 |
|  |  |  | **1.07** (0.94-1.22), **0.32** | | | | | | **1.10** (1.06-1.14), **2.04x10^-6^** | | | | | |
| ***STC1*** | rs17786744 | A | 0.17 | 0.13 | 0.48 | 0.48 | 0.35 | 0.39 | 0.17 | 0.17 | 0.48 | 0.50 | 0.35 | 0.34 |
|  |  |  | **1.19** (1.06-1.35), **0.01** | | | | | | **0.97** (0.94-1.01), **0.11** | | | | | |
| ***HNF4G*** | rs2941484 | T | 0.31 | 0.28 | 0.49 | 0.50 | 0.20 | 0.22 | 0.31 | 0.30 | 0.49 | 0.49 | 0.20 | 0.21 |
|  |  |  | **1.08** (0.96-1.21), **0.22** | | | | | | **1.06** (1.02-1.09), **3.24x10^-3^** | | | | | |
| ***ASAH2*** | rs10821905 | A | 0.68 | 0.69 | 0.29 | 0.28 | 0.03 | 0.04 | 0.68 | 0.65 | 0.29 | 0.31 | 0.03 | 0.04 |
|  |  |  | **0.99** (0.85-1.16), **0.93** | | | | | | **1.13** (1.08-1.180), **1.25x10^-7^** | | | | | |
| ***LTBP3*** | rs642803 | T | 0.29 | 0.31 | 0.50 | 0.49 | 0.22 | 0.21 | 0.29 | 0.31 | 0.50 | 0.50 | 0.22 | 0.19 |
|  |  |  | **0.95** (0.84-1.07), **0.38** | | | | | | **0.91** (0.88-0.94), **8.88x10^-8^** | | | | | |
| ***PTPN11*** | rs653178 | T | 0.24 | 0.26 | 0.50 | 0.48 | 0.27 | 0.26 | 0.23 | 0.26 | 0.50 | 0.49 | 0.27 | 0.25 |
|  |  |  | **0.93** (0.83-1.05), **0.26** | | | | | | **0.91** (0.88-0.95), **4.63x10^-7^** | | | | | |
| ***NRG4*** | rs1394125 | A | 0.41 | 0.40 | 0.46 | 0.46 | 0.13 | 0.15 | 0.41 | 0.39 | 0.46 | 0.47 | 0.13 | 0.15 |
|  |  |  | **1.04** (0.92-1.17), **0.56** | | | | | | **1.08** (1.04-1.12), **3.32x10^-5^** | | | | | |
| ***IGF1R*** | rs6598541 | A | 0.42 | 0.40 | 0.46 | 0.46 | 0.13 | 0.14 | 0.42 | 0.39 | 0.46 | 0.48 | 0.13 | 0.13 |
|  |  |  | **1.07** (0.95-1.21), **0.25** | | | | | | **1.07** (1.03-1.11), **4.25x10^-4^** | | | | | |
| ***NFAT5*** | rs7193778 | T | 0.02 | 0.02 | 0.25 | 0.27 | 0.73 | 0.71 | 0.02 | 0.03 | 0.25 | 0.28 | 0.73 | 0.70 |
|  |  |  | **0.96** (0.82-1.13), **0.64** | | | | | | **0.87** (0.83-0.92), **2.28x10^-8^** | | | | | |
| ***MAF*** | rs7188445 | A | 0.46 | 0.47 | 0.44 | 0.45 | 0.11 | 0.08 | 0.45 | 0.47 | 0.44 | 0.43 | 0.11 | 0.11 |
|  |  |  | **0.93** (0.82-1.06), **0.28** | | | | | | **0.97** (0.93-1.00), **0.07** | | | | | |
| ***HLF*** | rs7224610 | A | 0.16 | 0.17 | 0.48 | 0.49 | 0.37 | 0.34 | 0.16 | 0.18 | 0.48 | 0.48 | 0.37 | 0.34 |
|  |  |  | **0.91** (0.81-1.03), **0.13** | | | | | | **0.92** (0.89-0.96), **8.80x10^-6^** | | | | | |
| ***C17ORF82*** | rs2079742 | T | 0.02 | 0.02 | 0.24 | 0.24 | 0.74 | 0.74 | 0.02 | 0.02 | 0.24 | 0.23 | 0.75 | 0.76 |
|  |  |  | **1.01** (0.85-2.00), **0.92** | | | | | | **1.07** (1.01-1.13), **0.01** | | | | | |
| ***PRPSAP1*** | rs164009 | A | 0.15 | 0.12 | 0.48 | 0.50 | 0.37 | 0.38 | 0.15 | 0.15 | 0.48 | 0.47 | 0.37 | 0.38 |
|  |  |  | **1.09** (0.96-1.23), **0.17** | | | | | | **1.03** (1.00-1.07), **0.10** | | | | | |
| ***Number of loci associated with gout at experiment-wide significance*** | | | **4** | | | | | | **22** | | | | | |

Allelic odds ratios are used to report association data. Data are stratified by sex. CI, Confidence interval; Freq, frequency; SNP, single nucleotide polymorphism. Data are adjusted for age, body mass index, diuretic use and renal failure. Experiment wide-significance is defined as P < 0.0017.

**Table S4: Association and interaction between serum urate-associated single nucleotide polymorphisms and sex for gout risk, excluding pre-menopausal women**

| **Gene** | **SNP** | **Effect allele** | **Post-menopausal women**  **n=142272** | | **Men**  **n=171655** | | **Gene-sex interaction**  **P** |
| --- | --- | --- | --- | --- | --- | --- | --- |
|  |  |  | Risk allele absent  **Referent OR** | Risk allele present  **OR** (95% CI) | Risk allele absent  **OR** (95% CI) | Risk allele present  **OR** (95% CI) |  |
| **Loci replicated by Köttgen** | | | | | | | |
| ***ABCG2*** | rs2231142 | T | **1** | **1.60** (1.33-1.93) | **10.91** (9.81-12.13) | **26.06** (23.34-29.08) | **5.21x10^-5^** |
| ***SLC2A9*** | rs12498742 | A | **1** | **4.51** (2.14-9.52) | **18.02** (8.41-38.60) | **55.37** (26.36-116.33) | **0.33** |
| ***GCKR*** | rs1260326 | T | **1** | **1.39** (1.15-1.67) | **12.40** (10.55-14.58) | **17.15** (14.64-20.09) | **0.96** |
| ***SLC17A3*** | rs1165151 | T | **1** | **0.77** (0.65-0.92) | **12.09** (10.41-14.03) | **9.70** (8.39-11.23) | **0.70** |
| ***SLC22A11*** | rs478607 | A | **1** | **0.76** (0.46-1.23) | **12.28** (7.42-20.34) | **9.30** (5.74-15.07) | **0.99** |
| ***PDZK1*** | rs1471633 | A | **1** | **0.92** (0.76-1.10) | **9.60** (8.17-11.29) | **12.40** (10.60-14.50) | **5.02x10^-4^** |
| ***INHBE*** | rs3741414 | T | **1** | **0.80** (0.67-0.95) | **12.23** (10.93-13.68) | **9.97** (8.89-11.18) | **0.81** |
| ***SLC16A9*** | rs1171614 | T | **1** | **0.94** (0.79-1.12) | **12.97** (11.58-14.54) | **10.67** (9.49-11.99) | **0.15** |
| ***SLC22A11*** | rs2078267 | T | **1** | **0.75** (0.62-0.91) | **11.99** (10.06-14.29) | **9.33** (7.86-11.06) | **0.71** |
| ***RREB1*** | rs675209 | T | **1** | **1.27** (1.07-1.51) | **13.27** (11.67-15.08) | **14.62** (12.86-16.63) | **0.11** |
| **Loci reported by Köttgen** | | | | | | | |
| ***PKLR*** | rs11264341 | T | **1** | **0.80** (0.67-0.95) | **11.74** (10.16-13.55) | **10.16** (8.82-11.70) | **0.39** |
| ***INHBB*** | rs17050272 | A | **1** | **1.07** (0.89-1.28) | **12.72** (10.92-14.83) | **12.98** (11.17-15.09) | **0.62** |
| ***ACVR2A*** | rs2307394 | T | **1** | **1.00** (0.75-1.33) | **13.51** (10.13-18.01) | **12.19** (9.23-16.09) | **0.51** |
| ***MUSTN1*** | rs6770152 | T | **1** | **0.97** (0.77-1.20) | **13.97** (11.36-17.19) | **11.65** (9.53-14.26) | **0.21** |
| ***TMEM171*** | rs17632159 | C | **1** | **0.75** (0.63-0.89) | **11.57** (10.26-13.04) | **10.21** (9.06-11.52) | **0.07** |
| ***VEGFA*** | rs729761 | T | **1** | **0.96** (0.81-1.14) | **12.45** (10.99-14.10) | **11.68** (10.30-13.23) | **0.82** |
| ***MLXIPL*** | rs1178977 | A | **1** | **1.62** (0.95-2.76) | **15.58** (9.04-26.86) | **19.85** (11.72-33.62) | **0.39** |
| ***PRKAG2*** | rs10480300 | T | **1** | **0.97** (0.82-1.16) | **11.63** (10.29-13.14) | **12.85** (11.37-14.52) | **0.17** |
| ***STC1*** | rs17786744 | A | **1** | **1.32** (1.03-1.70) | **15.68** (12.31-19.96) | **15.72** (12.42-19.90) | **0.04** |
| ***HNF4G*** | rs2941484 | T | **1** | **1.18** (0.98-1.44) | **13.41** (11.30-15.92) | **14.15** (11.97-16.74) | **0.26** |
| ***ASAH2*** | rs10821905 | A | **1** | **0.95** (0.79-1.15) | **11.69** (10.49-13.03) | **13.31** (11.90-14.88) | **0.07** |
| ***LTBP3*** | rs642803 | T | **1** | **0.88** (0.74-1.06) | **12.27** (10.46-14.39) | **10.95** (9.37-12.80) | **0.93** |
| ***PTPN11*** | rs653178 | T | **1** | **0.85** (0.70-1.04) | **12.21** (10.26-14.52) | **10.57** (8.92-12.51) | **0.89** |
| ***NRG4*** | rs1394125 | A | **1** | **1.00** (0.84-1.19) | **11.77** (10.23-13.53) | **12.76** (11.12-14.64) | **0.39** |
| ***IGF1R*** | rs6598541 | A | **1** | **1.04** (0.87-1.23) | **11.77** (10.24-13.53) | **13.05** (11.38-14.98) | **0.46** |
| ***NFAT5*** | rs7193778 | T | **1** | **1.28** (0.68-2.40) | **21.05** (11.10-39.91) | **15.54** (8.33-28.99) | **0.10** |
| ***MAF*** | rs7188445 | A | **1** | **0.98** (0.83-1.17) | **12.58** (11.04-14.33) | **11.91** (10.46-13.56) | **0.67** |
| ***HLF*** | rs7224610 | A | **1** | **0.90** (0.72-1.14) | **12.66** (10.18-15.74) | **11.17** (9.04-13.79) | **0.84** |
| ***C17ORF82*** | rs2079742 | T | **1** | **0.95** (0.52-1.74) | **9.74** (5.20-18.27) | **11.81** (6.50-21.45) | **0.46** |
| ***PRPSAP1*** | rs164009 | A | **1** | **1.31** (1.01-1.71) | **15.40** (11.94-19.87) | **15.64** (12.20-20.05) | **0.06** |

Association and interaction data are reported according to effect allele presence or absence with pre-menopausal women excluded from the analysis. CI, confidence interval; OR, odds ratio; SNP, single nucleotide polymorphism. Data are adjusted by age, body mass index, diuretic use and renal failure. Experiment wide-significance is defined as P < 0.0017.

**Table S5: Association and interaction between serum urate-associated single nucleotide polymorphisms and sex for gout risk according to the number of effect alleles present**

| **Gene** | **SNP** | **Effect allele** | **Women * effect allele number**  **OR**  **n=188221** | | | **Men * effect allele number**  **OR**  **n=171655** | | | **Gene-sex interaction**  **P** |
| --- | --- | --- | --- | --- | --- | --- | --- | --- | --- |
|  |  |  | **0 effect alleles**  **(Referent)** | **1 effect allele** | **2 effect alleles** | **0 effect alleles** | **1 effect allele** | **2 effect alleles** |  |
| **Loci replicated by Köttgen** | | | | | | | | | |
| ***ABCG2*** | rs2231142 | T | **1** | **1.56** (1.29-1.88) | **2.48** (1.50-4.11) | **11.99** (10.81-13.30) | **26.95** (24.18-30.03) | **56.65** (48.16-66.63) | **1.47x10^-4^** |
| ***SLC2A9*** | rs12498742 | A | **1** | **2.36** (1.15-4.82) | **5.21** (2.58-10.49) | **18.10** (8.85-37.00) | **39.13** (19.52-78.44) | **65.98** (32.94-132.14) | **0.03** |
| ***GCKR*** | rs1260326 | T | **1** | **1.35** (1.11-1.63) | **1.64** (1.29-2.08) | **13.80** (11.77-16.18) | **18.15** (15.52-21.22) | **21.99** (18.69-25.88) | **0.97** |
| ***SLC17A3*** | rs1165151 | T | **1** | **0.77** (0.64-0.93) | **0.75** (0.59-0.95) | **13.15** (11.38-15.20) | **11.15** (9.66-12.86) | **9.13** (7.85-10.62) | **0.33** |
| ***SLC22A12*** | rs478607 | A | **1** | **0.84** (0.51-1.39) | **0.76** (0.47-1.25) | **13.96** (8.44-23.10) | **11.79** (7.27-19.11) | **10.15** (6.27-16.43) | **0.84** |
| ***PDZK1*** | rs1471633 | A | **1** | **0.94** (0.78-1.14) | **0.87** (0.68-1.10) | **10.54** (9.00-12.34) | **13.02** (11.17-15.19) | **14.97** (12.78-17.52) | **3.29x10^-4^** |
| ***INHBE*** | rs3741414 | T | **1** | **0.84** (0.70-1.00) | **0.71** (0.48-1.06) | **13.58** (12.16-15.15) | **11.53** (10.29-12.91) | **8.25** (7.02-9.69) | **0.72** |
| ***SLC16A9*** | rs1171614 | T | **1** | **0.97** (0.81-1.16) | **0.81** (0.53-1.22) | **14.28** (12.78-15.96) | **12.13** (10.81-13.61) | **9.18** (7.77-10.84) | **0.26** |
| ***SLC22A11*** | rs2078267 | T | **1** | **0.78** (0.63-0.96) | **0.79** (0.63-0.99) | **13.59** (11.42-16.17) | **11.41** (9.63-13.53) | **9.22** (7.75-10.96) | **0.11** |
| ***RREB1*** | rs675209 | T | **1** | **1.22** (1.02-1.44) | **1.29** (0.95-1.75) | **14.28** (12.62-16.16) | **15.64** (13.80-17.73) | **16.27** (14.03-18.87) | **0.48** |
| **Loci reported by Köttgen** | | | | | | | | | |
| ***PKLR*** | rs11264341 | T | **1** | **0.83** (0.69-1.00) | **0.79** (0.62-1.01) | **13.09** (11.37-15.08) | **11.70** (10.17-13.45) | **10.37** (8.94-12.04) | **0.72** |
| ***INHBB*** | rs17050272 | A | **1** | **1.12** (0.93-1.34) | **1.07** (0.83-1.36) | **14.25** (12.26-16.57) | **14.27** (12.30-16.56) | **15.32** (13.11-17.90) | **0.44** |
| ***ACVR2A*** | rs2307394 | T | **1** | **1.05** (0.79-1.42) | **0.93** (0.70-1.26) | **14.72** (11.13-19.48) | **13.29** (10.13-17.43) | **13.28** (10.13-17.42) | **0.35** |
| ***MUSTN1*** | rs6770152 | T | **1** | **1.02** (0.82-1.28) | **0.87** (0.68-1.11) | **15.29** (12.50-18.70) | **13.20** (10.84-16.08) | **12.09** (9.91-14.75) | **0.35** |
| ***TMEM171*** | rs17632159 | C | **1** | **0.74** (0.62-0.89) | **0.87** (0.65-1.18) | **12.81** (11.39-14.41) | **11.49** (10.20-12.94) | **10.523** (9.13-12.13) | **0.09** |
| ***VEGFA*** | rs729761 | T | **1** | **0.98** (0.82-1.17) | **1.04** (0.76-1.42) | **13.89** (12.29-15.70) | **13.12** (11.60-14.85) | **12.60** (10.86-14.61) | **0.70** |
| ***MLXIPL*** | rs1178977 | A | **1** | **1.38** (0.80-2.40) | **1.87** (1.09-3.18) | **17.97** (10.42-30.97) | **20.16** (11.89-34.20) | **24.23** (14.30-41.05) | **0.30** |
| ***PRKAG2*** | rs10480300 | T | **1** | **0.88** (0.74-1.06) | **1.45** (1.10-1.91) | **12.74** (11.32-14.35) | **13.77** (12.21-15.52) | **15.71** (13.63-18.09) | **0.03** |
| ***STC1*** | rs17786744 | A | **1** | **1.28** (0.99-1.66) | **1.47** (1.13-1.92) | **17.59** (13.86-22.33) | **18.19** (14.40-22.97) | **16.90** (13.36-21.36) | **5.23x10^-3^** |
| ***HNF4G*** | rs2941484 | T | **1** | **1.09** (0.90-1.33) | **1.15** (0.90-1.46) | **14.00** (11.89-16.49) | **14.41** (12.27-16.93) | **15.67** (13.27-18.51) | **0.85** |
| ***ASAH2*** | rs10821905 | A | **1** | **0.94** (0.78-1.13) | **1.17** (0.75-1.82) | **12.86** (11.57-14.29) | **14.41** (12.90-16.09) | **16.71** (14.21-19.66) | **0.19** |
| ***LTBP3*** | rs642803 | T | **1** | **0.93** (0.77-1.13) | **0.90** (0.71-1.14) | **13.81** (11.80-16.16) | **12.77** (10.94-14.90) | **11.33** (9.65-13.31) | **0.73** |
| ***PTPN11*** | rs653178 | T | **1** | **0.84** (0.69-1.02) | **0.86** (0.69-1.08) | **13.30** (11.24-15.74) | **11.73** (9.95-13.84) | **11.12** (9.40-13.16) | **0.73** |
| ***NRG4*** | rs1394125 | A | **1** | **1.00** (0.84-1.19) | **1.11** (0.86-1.43) | **13.07** (11.40-14.98) | **13.81** (12.06-15.82) | **15.45** (13.35-17.88) | **0.81** |
| ***IGF1R*** | rs6598541 | A | **1** | **1.02** (0.85-1.22) | **1.19** (0.92-1.54) | **13.05** (11.38-14.96) | **14.47** (12.64-16.58) | **14.49** (12.50-16.79) | **0.45** |
| ***NFAT5*** | rs7193778 | T | **1** | **1.18** (0.65-2.13) | **1.09** (0.61-1.95) | **20.20** (11.30-36.34) | **16.24** (9.18-28.73) | **14.45** (8.17-25.53) | **0.37** |
| ***MAF*** | rs7188445 | A | **1** | **1.01** (0.85-1.20) | **0.77** (0.57-1.05) | **13.64** (12.01-15.48) | **12.89** (11.35-14.64) | **13.01** (11.28-15.02) | **0.24** |
| ***HLF*** | rs7224610 | A | **1** | **0.94** (0.74-1.19) | **0.84** (0.66-1.08) | **13.76** (11.13-17.00) | **12.52** (10.19-15.39) | **11.63** (9.45-14.30) | **0.94** |
| ***C17ORF82*** | rs2079742 | T | **1** | **0.91** (0.50-1.65) | **0.92** (0.52-1.65) | **10.34** (5.65-18.93) | **12.03** (6.78-21.32) | **12.70** (7.17-22.47) | **0.64** |
| ***PRPSAP1*** | rs164009 | A | **1** | **1.34** (1.03-1.76) | **1.29** (0.98-1.70) | **17.01** (13.25-21.82) | **16.85** (13.21-21.50) | **17.82** (13.95-22.75) | **0.09** |

Association and interaction data are reported according to the number of effect alleles. CI, confidence interval; OR, odds ratio; SNP, single nucleotide polymorphism. Data are adjusted by age, body mass index, diuretic use and renal failure. Experiment wide-significance is defined as P < 0.0017.
