# Supplementary figures and images for: Interactions between serum urate-associated genetic variants and sex on gout risk: analysis of the UK Biobank
Source: Arthritis Res Ther. 2019 Jan 9;21:13. doi: 10.1186/s13075-018-1787-5 (PMC6327586; doi:10.1186/s13075-018-1787-5)

## Slide 1
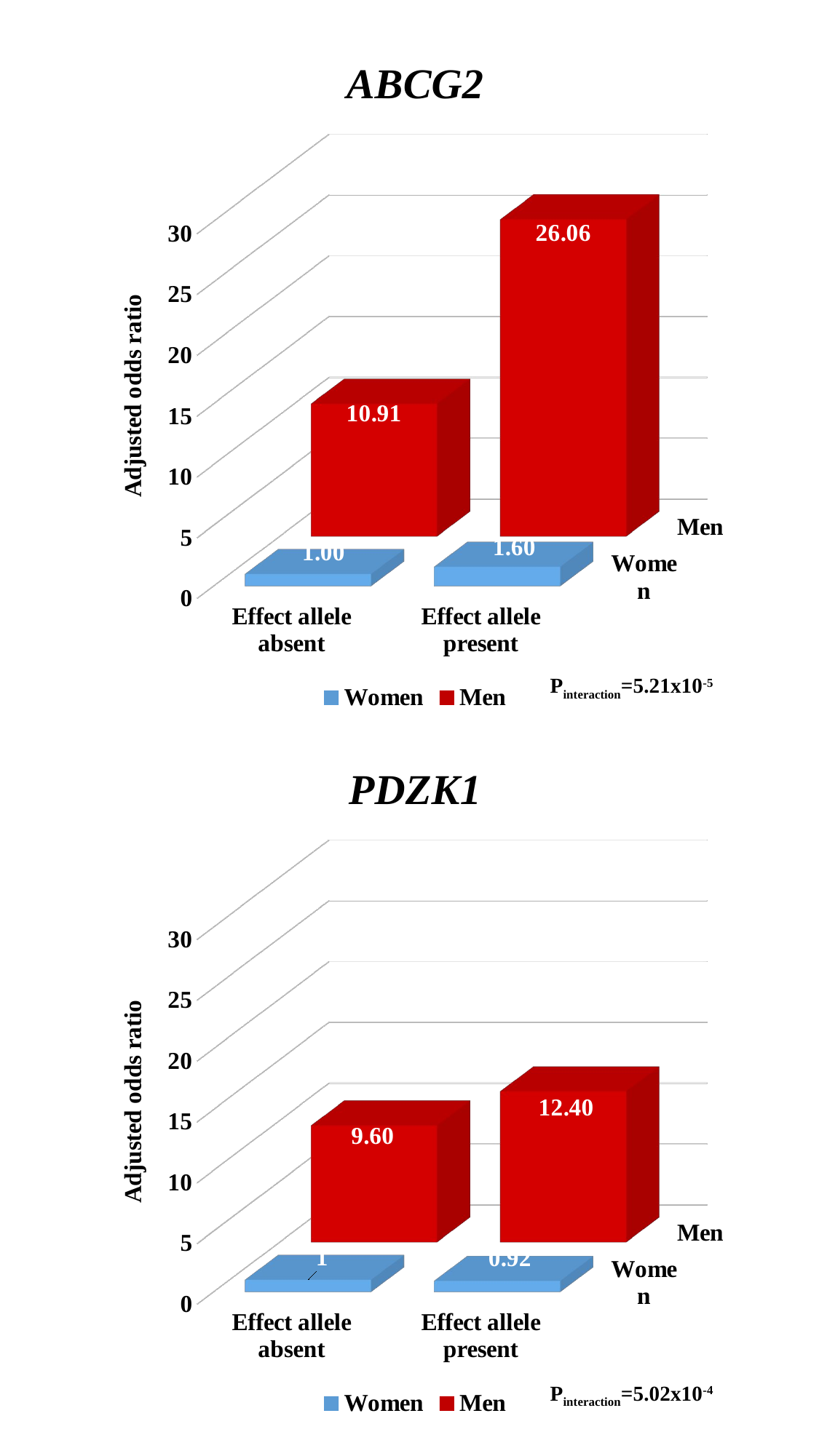

[unsupported chart]
Pinteraction=5.21x10-5
[unsupported chart]
Pinteraction=5.02x10-4

Supplement: Supplementary file 2 — Figure S1. Association and interaction between serum urate-associated genetic variants (for ABCG2 and PDZK1) and sex for gout risk according to effect allele presence, excluding pre-menopausal women. Data are adjusted for age, body mass index, renal failure, and diuretic use. Experiment-wide significance is defined as P < 0.0017. (PPTX 44 kb) [file 13075_2018_1787_MOESM2_ESM.pptx]
